# Influence of agro‐environmental pollutants on a biocontrol strain of *Bacillus velezensis*

**DOI:** 10.1002/mbo3.660

**Published:** 2018-06-25

**Authors:** Mónika Vörös, László Manczinger, László Kredics, András Szekeres, Kadaikunnan Shine, Naiyf S. Alharbi, Jamal M. Khaled, Csaba Vágvölgyi

**Affiliations:** ^1^ Department of Microbiology Faculty of Science and Informatics University of Szeged Szeged Hungary; ^2^ Department of Botany and Microbiology College of Science King Saud University Riyadh Saudi Arabia

**Keywords:** *Bacillus*, biocontrol, enzymes, heavy metals, pesticides

## Abstract

Metal‐ and pesticide‐tolerant biocontrol agents are preferred in integrated pest management, as such strains can be applied in combination with different pesticides. The *Bacillus velezensis* strain SZMC 6161J proved to be sensitive to copper, nickel, zinc, and cadmium, while manganese elevated its growth. At concentrations higher than 1 mmol L^−1^, zinc and iron inhibited the chymotrypsin‐like activity of this strain. In addition, trypsin‐like protease and palmitoyl esterase activities were insensitive to all tested heavy metals in the applied concentration range. We studied the effects of some widely used herbicides and fungicides on the growth of this strain. The presence of sulfonylurea herbicides, like bensulfuron‐methyl, cinosulfuron, chlorsulfuron, ethoxysulfuron, triasulfuron, and primisulfuron‐methyl strongly inhibited the biomass production of the strain even at the concentration of 6.25 mg L^−1^. Glyphosate also inhibited the growth above 30 mg L^−1^. Similarly, contact fungicides like captan, maneb, mancozeb, and thiram resulted in total inhibition at the concentration as low as 6.25 mg L^−1^. Interestingly, the sterol‐biosynthesis‐inhibiting fungicides imazalil, fenarimol, penconazole, and tebuconazole also proved to be potent inhibitors. Heavy metal‐ and fungicide‐tolerant strains were isolated from the parental strain and their antagonistic abilities were evaluated. There was no substantial difference between the antagonism capability of wild‐type strain and the resistant mutants.

## 1 INTRODUCTION

Certain microorganisms cause serious pre‐ and postharvest damages with detrimental effects leading to the reduction in the amount and quality of various crops. The most frequently used approach for controlling these plant diseases is the application of synthetic pesticides for the treatment of plants and their environments (Strange, 1993). However, certain crops, particularly monocultures, require continuously increasing doses of these chemicals, generating a dangerous base for the emergence of resistant plant pathogens. Due to their ecotoxicological properties, xenobiotics can also damage the balanced structure of microbial communities in the soil. When xenobiotics resist biological degradation and persist in the soil for an extended period of time, these compounds may enter the food chain and cause toxic effects to animals and humans (Cawoy, Bettiol, Fickers, & Ongena, 2011; Peng, Shandong, Chen, & Zhou, 2013; Russell, 1995). Thus, there is an increasing demand for new approaches in plant protection to achieve control over diseases and minimize the negative impacts on humans and the environment (NRC Report, 1996; Emmert & Handelsman, 1999). Currently, organic farming practices like crop rotation, application of green manure, composting, biological pest control, and integrated pest management are important techniques for sustainable “green” agriculture (Doyle, 1997). Among these techniques, biological control is expected to have an increasing importance for the reduction in plant diseases in the future (Emmert & Handelsman, 1999; Peng et al., 2013). Biocontrol agents (BCAs) are environmentally friendly and can remarkably control plant diseases, but these agents are frequently sensitive to the effect of various environmental factors. In most cases, BCAs are also less efficient than chemical control agents (Peng et al., 2013). Therefore, a good BCA is expected to display wide tolerance to be able to grow and produce antibiotics and/or extracellular enzymes at changing values of environmental parameters such as temperature, pH of soil, water availability, and soil pollutants such as various metals (particularly Cu, Ni, Cd, Zn, Cr, and Pb) (Effron, de la Horra, Defrieri, Fontanive, & Palma, 2004) and chemicals used in plant protection.

Different mechanisms form the basis of biological pest control, for example, competition for nutrients and/or the ecological niche in the rhizosphere, production of inhibitory molecules (antibiotics or extracellular enzymes), parasitism, or the induction of systemic resistance in host plants (Pal & Gardener, 2006; Winding, Binnerup, & Pritchard, 2004). The most important extracellular enzymes of disease‐suppressive bacteria include proteases, cellulases, β‐1,3‐glucanases, chitinases, and lipases. Some of these enzymes (e.g., chitinases) are directly antagonistic to fungi, while others have importance in the competition processes for the carbon and nitrogen sources as well as for the rhizosphere competence.

Among bacteria, the genera *Agrobacterium, Pseudomonas, Bacillus, Alcaligenes,* and *Streptomyces* include strains with outstanding biocontrol properties (Shoda, 2000). The gram‐positive *Bacillus* species have high thermotolerance, are typically present in all types of soil, can rapidly grow in liquid culture, and easily form resistant endospores. Most of these bacteria are generally regarded as safe; therefore, their application as BCAs is frequently considered (Shoda, 2000). Consequently, several *Bacillus* strains are used in the practice; particularly as these microorganisms frequently show strong antagonistic effects against plant pathogenic fungi (Kim, Song, Lee, Yeo, & Yun, 2013).

Protection against plant pathogenic fungi and weeds are crucial components of modern agricultural technologies. Despite this, the traditional pesticide‐based agriculture has its limitations: resistance may develop in the target organisms to the pesticides, and/or the xenobiotics may be harmful for the natural ecosystems and human health. New agricultural approaches like organic farming or integrated pest management (IPM) try to overcome these limitations. Generally, biocontrol is a key element in both technologies as a series of different plant diseases can be controlled by well‐selected fungal and/or bacterial antagonists (Brannen & Kenney, 1997). The coapplication of a fungicide in combination with BCAs significantly enhances the efficiency of disease control compared to treatments with BCAs alone. Boukaew, Klinmanee, and Prasertsan (2013) reported that the combined application of *Streptomyces philanthi* RM‐1‐138 with carbendazim provided better control of sheath blight in rice caused by *Rhizoctonia solani*, than the separate application of the BCA or the fungicide. IPM is an eco‐friendly alternative agricultural approach (Boukaew et al., 2013; Shaw, 1982), in which biological, chemical, and physical means of control, resistant crop cultivars, and modification of cultural practices are combined to give stable, long‐term protection, and prevent or reduce pest infestations (Doyle, 1997).

The ability of BCAs to germinate rapidly and in large numbers is necessary for effective pest control. However, they could be sensitive to the different xenobiotics used in the agriculture. In these cases, the simultaneous application of a BCA and an inhibitory pesticide is contraindicated. Numerous *Bacillus* strains have been used as BCAs by Jacobsen, Zidack, and Larson (2004), while others are intensively studied as possible BCAs (Pane & Zaccardelli, 2015). They can produce a wide range of different secondary metabolites, antibiotics, and extracellular enzymes, which are important in their interactions with the pathogens. The extracellular enzymes produced by these bacteria can contribute to their antagonistic and competition efficacy. Therefore, the effects of various metals and other agro‐environmental pollutants on the activity of the secreted extracellular enzymes of bacilli are worth examining.

The aim of this study was to investigate the effect of distinct agro‐environmental pollutants on the growth and enzyme activities of *B. velezensis* strain SZMC 6161J isolated from tomato rhizosphere (Vágvölgyi et al., 2013) and to determine its main ecophysiological properties and spectrum of antagonism. In a previous publication, we investigated *Bacillus*,* Pseudomonas*, and *Pantoea* isolates which showed excellent antagonistic abilities against phytopathogenic microorganisms and produced many types of extracellular enzymes (e.g., proteases, chitinases, cellobiohydrolases, lipases, and β‐glucosidases) (Vágvölgyi et al., 2013). Besides possessing a wide antagonistic spectrum, one of the isolates, *B. velezensis* SZMC 6161J revealed outstanding chymotrypsin‐like, trypsin‐like, and esterase activities. The exoenzyme activities of this strain were further examined in this study. The taxonomic position of the strain was determined through partial sequencing of the *gyrA* gene (GenBank accession number JX683905.1) (Vágvölgyi et al., 2013). In the present work the ecophysiological properties of this strain were also explored and its antagonistic spectrum was reexamined.

## 2 MATERIALS AND METHODS

### 2.1 Bacterial strain and growth conditions

The *B. velezensis* strain SZMC 6161J isolated from the rhizosphere of a tomato plant (Vágvölgyi et al., 2013) and deposited in the Szeged Microbiology Collection (SZMC; Szeged, Hungary; www.szmc.hu) was used in this study. The strain was cultured on yeast‐glucose (YEG) medium (glucose 0.2%, yeast extract 0.2%, bacto agar 2%) at 25°C.

### 2.2 Pesticides

Fungicides such as captan, carbendazim, carboxine, fenarimol, flutriafol, imazalil, mancozeb, maneb, penconazole, tebuconazole, thiabendazole, thiram, and thiophanate‐methyl, or herbicides such as bensulfuron‐methyl, cinosulfuron, chlorsulfuron, chlortoluron, diuron, ethoxysulfuron, glyphosate, isoproturon, linuron, primisulfuron‐methyl, propham, triasulfuron, and 2,4‐dichlorophenoxyacetic acid (2,4‐D) were used in the experiments. All compounds were purchased from Sigma‐Aldrich (Hungary). For stock solutions, the pesticides were dissolved in 96% ethanol at 2 mg ml^−1^ concentrations.

### 2.3 Effect of heavy metals on bacterial growth

For investigation of the effect of heavy metals on the growth of *B. velezensis* SZMC 6161J, the strain was grown in liquid YEG medium in the presence of heavy metals at 25°C for 5 days. The metal salts (CuSO_4_, MnSO_4_, NiSO_4_, FeSO_4,_ ZnSO_4_, and CdSO_4_) were tested at the concentrations of 0.1 mmol L^−1^, 0.5 mmol L^−1^, and 1 mmol L^−1^. After incubation, the optical density of the cultures was measured at 620 nm using a microplate spectrophotometer (SPECTROstar Nano; BMG Labtech, Offenburg, Germany). The experiments were performed in triplicate.

### 2.4 Effects of pesticides on the growth of *B*. *velezensis*

Inhibition of bacterial growth was examined in shaken minicultures (2.5 ml medium volume) at 25°C with Besson's liquid minimal medium (Besson, Chevanet, & Michel, 1987) supplemented with 5 mg L^−1^ MnSO_4_ and with pesticides from the stock solutions to 6.25, 12.5, and 25 mg L^−1^ end concentrations. The liquid media were inoculated with 10^5^ CFU ml^−1^ of *B. velezensis* SZMC 6161J cultured overnight at 140 rpm in a rotary shaker (IKA KS 4000 IC Control). Cell density of the cultures was determined by turbidimetry at 620 nm after 48 hr. Experiments were performed in triplicate.

### 2.5 Enzyme measurements from liquid cultures produced in the presence of distinct heavy metals

Shaken liquid cultures were produced in 100‐ml Erlenmeyer flasks containing 30 ml of liquid medium and shaken in a rotary shaker (IKA KS 4000 IC Control, 150 rpm). YEG liquid medium was used in the presence of heavy metals at 25°C for 5 days. The metal salts (CuSO_4_, MnSO_4_, NiSO_4_, FeSO_4,_ ZnSO_4_, and CdSO_4_) were tested at the concentrations of 0.1, 0.5, and 1 mmol L^−1^. Protease and esterase enzyme activities were measured using chromogenic substrates. *N*‐succinyl‐l‐Ala‐l‐Ala‐l‐Pro‐l‐Phe‐p‐nitroanilide and *N*‐Bz‐l‐Phe‐l‐Val‐l‐Arg‐p‐nitroanilide were used for the determination of extracellular chymotrypsin‐like and trypsin‐like protease activities, respectively, while p‐nitrophenyl‐palmitate was used for the determination of extracellular esterase activities. Secreted enzymes were measured from the cell‐free supernatants of shaken liquid cultures after 5 days of culturing. The cells were pelleted at 10,000 g for 5 min, and subsequently the supernatant was analyzed using chromogenic substrates: 50 μl of phosphate buffer (pH 6.6) and 50 μl chromogenic substrate (3 mmol L^−1^, dissolved in dimethylsulfoxide) were mixed in the wells of a sterile microtiter plate, and 50 μl amounts of the supernatants were added. The final concentration of the substrates was 1 mmol L^−1^. These mixtures were incubated at 25°C for 20 min, and the formed (yellow) reaction product was quantified in a microtiter plate spectrophotometer (SPECTROstar Nano; BMG Labtech, Offenburg, Germany) at 405 nm. These experiments were performed in triplicate.

### 2.6 Effect of heavy metals on enzyme activities in cell‐free supernatants of shaken liquid cultures produced in heavy metal‐free medium

The heavy metal compounds were added to the cell‐free supernatants of shaken YEG liquid cultures to the end concentrations of 0.125, 0.25, 0.5, 1, 2, and 4 mmol L^−1^. The samples were incubated at 25°C for 30 min and subsequently 50 μl phosphate buffer (pH 6.6) and 50 μl chromogenic substrate (final concentration: 1 mmol L^−1^) were added. After incubation at 25°C for 20 min, the yellow reaction product was quantified using a microtiter plate spectrophotometer (SPECTROstar Nano; BMG Labtech, Offenburg, Germany) at 405 nm. The experiments were performed in triplicate.

### 2.7 Isolation and characterization of heavy metal‐ and pesticide‐tolerant mutants

After determination of the minimum inhibitory concentration (MIC) values for strain SZMC 6161J, spontaneous resistant clones were isolated by plating 10^7^ cells on YEG media supplemented with each agro‐environmental pollutant at their MIC levels. Colonies with the most intense growth were isolated and subcultured twice on the same supplemented medium.

The MIC values of mutants related to metals and pesticides (compared with the wild‐type strain), were determined in mirocultures established on microtiter plates with 96 sample wells. The reaction wells contained 0.2–0.2 ml YEG medium and the inhibitors in twofold serial dilutions. Media were inoculated with 0.05–0.05 ml, 24‐hr‐old cultures of the bacterial strains (end cell concentration was 10^5^ ml^−1^) and incubated at 25°C for 48 hr. Finally, the OD of the cultures was measured at 620 nm with a microplate reader. From these data, growth inhibitory curves were created with Microsoft Excel 2010 software and the exact MIC values were determined with extrapolation of the curves. The examination was performed in three parallels.

To investigate the stability of tolerant mutants, 10 randomly selected mutant strains were inoculated into 0.2–0.2 ml YEG medium (end cell concentration: 2.5 × 10^4^ ml^−1^) and incubated at 25°C for 24 hr. From each minicultures, 0.05 ml was transferred to 0.2 ml YEG medium and incubated again at 25°C for 24 hr. After the incubation, 10‐fold serial dilutions were made from these cultures, than from the 10^5^ and 10^6^ times diluted samples 0.05 ml was spread onto YEG plates, and YEG plates supplemented with the corresponding inhibitor at the isolation concentration. After 2 days incubation at 25°C, the colony numbers were determined. The stability of a mutant in percentage was calculated as follows: number of colonies on inhibitor containing plates divided with the number of colonies on the control YEG plates and multiplied with 100. The investigation was performed in three parallels. The statistical box plot graph showing the distribution of the mutant stability values of tolerant mutants was generated with the Gnumeric Spreadsheet 1.10.16 software.

### 2.8 Antagonism investigations with *B. velezensis* SZMC 6161J and its tolerant mutants against phytopathogenic bacteria and fungi

In vitro antagonism tests were performed to examine the growth inhibition of plant pathogenic bacteria. One milliliter of the pathogenic bacterial culture was gently mixed with 25 ml of slightly precooled (37–40°C) medium (0.2% yeast extract, 0.2% glucose, 1% agar), and poured into Petri dishes. The plates were dried in a sterile compartment and a 5‐mm‐diameter paper disk was placed on the surface of the culture medium. Five microliter suspension of overnight‐cultured *B. velezensis* SZMC 6161J (10^5^ CFU ml^−1^) was added to the paper disk. The plates were incubated at 25°C. Inhibition zones becoming visible after 24–48 hr of incubation were measured. The antagonism tests were performed in three parallels.

To evaluate the inhibition of plant pathogenic filamentous fungi, 8‐mm‐diameter mycelial disks were cut from the peripheral part of their 4‐ to 6‐day‐old colonies (grown on malt extract medium) and placed on the surface of YEG plates. After 48 hr of incubation, 5 μl of *B. velezensis* SZMC 6161J 24‐hr‐old shaken culture was spot‐inoculated, 40 mm apart from the previously inoculated fungus. Control plates containing only the test fungus were also prepared. Plates were incubated at 25°C until the colony radius of the control fungal colony reached 40 mm; this time varied depending on the growth characteristic of the examined fungus. The diameter of the inhibition zone between edge of the bacterium colony and the inhibited fungal colony was measured. The tests were performed in three parallels.

### 2.9 Statistical analyses

ANOVA statistical analyses were performed with the MedCalc 17.5.5. software.

## 3 RESULTS

### 3.1 Ecophysiological properties of strain *B. velezensis* SZMC 6161J

Strain *B. velezensis* SZMC 6161J has ecophysiological characters typical for *Bacillus*: its growth temperature optimum is around 40°C, the pH optimum is 7.9, and the minimal water activity for growth is 0.954. It proved to be an excellent antagonistic strain against *Agrobacterium, Erwinia*,* Xanthomonas*, and numerous fungal (*Alternaria, Botrytis, Colletotrichum, Fusarium Rhizoctonia*, and *Sclerotinia*) species (Vágvölgyi et al., 2013). The antifungal effect of the strain is due to the secreted distinct molecular forms of fengycin (a depsipeptide compound), which was proved with HPLC scan and MS‐MS analysis. Surfactins or iturins could not be detected in the cell‐free supernatants of shaken liquid cultures (results not shown).

*Bacillus velezensis* strains frequently produce large amounts of bacilysin, bacillaene, and difficidin (Chen et al., 2006, 2009) among which difficidin is a strong inhibitor of *Xanthomonas* and *Erwinia*. Antimicrobial spectrum of our *Bacillus* strain, with very efficient inhibition of *Erwinia* and *Xanthomonas* suggests that it also could be difficidin producer.

Furthermore, with TLC‐stamp bioautographic method of Chen et al. (2006), we detected very likely difficidin in YEG liquid culture of our strain which showed significant inhibition both against *Agrobacterium* and *Xanthomonas*, but other antibacterial compounds could not be detected in the culture of the strain *B. velezensis* SZMC 6161J (results not shown).

### 3.2 Effect of heavy metals on strain *B. velezensis* SZMC 6161J

**Figure 1:**
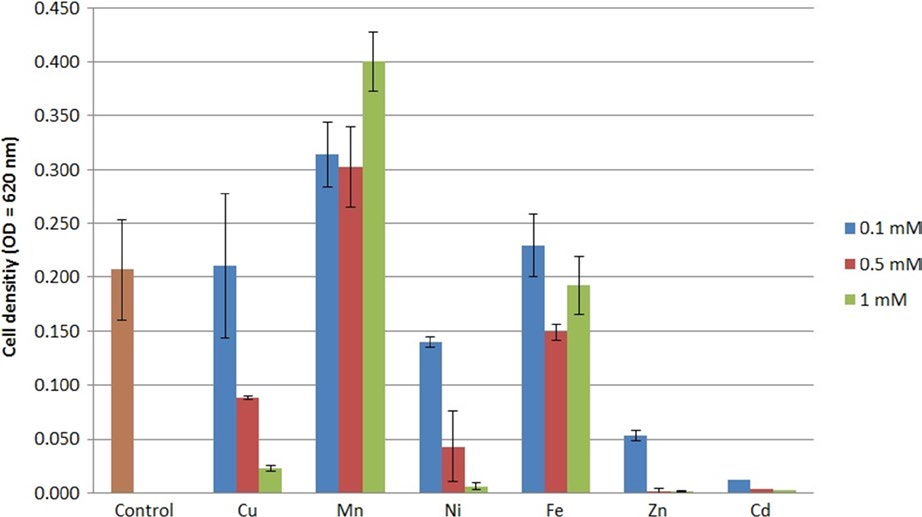
Effect of distinct heavy metal ions on the growth of *Bacillus velezensis* SZMC 6161J in YEG medium. Cell densities on the fifth day

**Figure 2:**
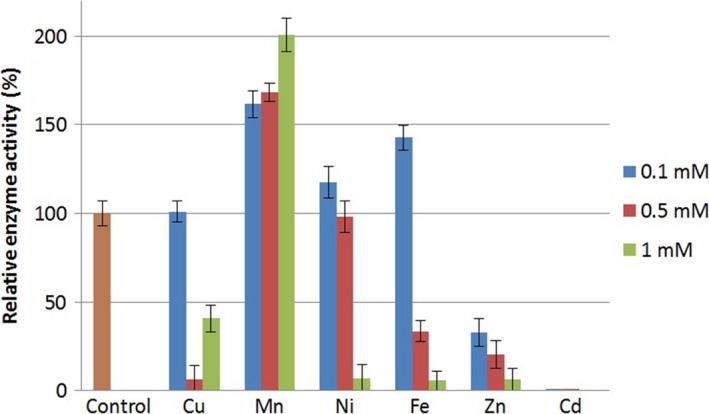
Measurable chymotrypsin‐like protease activities on the fifth day in the cell‐free supernatant of shaken liquid cultures of *B. velezensis* SZMC 6161J, produced in the presence of distinct heavy metal ions

The strain *B. velezensis* SZMC 6161J grew well in the presence of 0.1 mmol L^−1^ copper, manganese, nickel, and iron, but showed increased sensitivity to cadmium and zinc (Figure 1). Manganese elevated the growth of the strain. The secretion and activities of extracellular chymotrypsin‐like proteases were measured in the presence of different concentrations (0.1, 0.5 and 1 mmol L^−1^) of various metal salts. Cadmium, copper, nickel, iron, and zinc inhibited chymotrypsin‐like enzyme secretion (Figure 2). Increased activity of the secreted chymotrypsin‐like proteases was observed in the presence of manganese at all concentrations, and also in the presence of 0.1 mmol L^−1^ nickel and iron.

**Figure 3:**
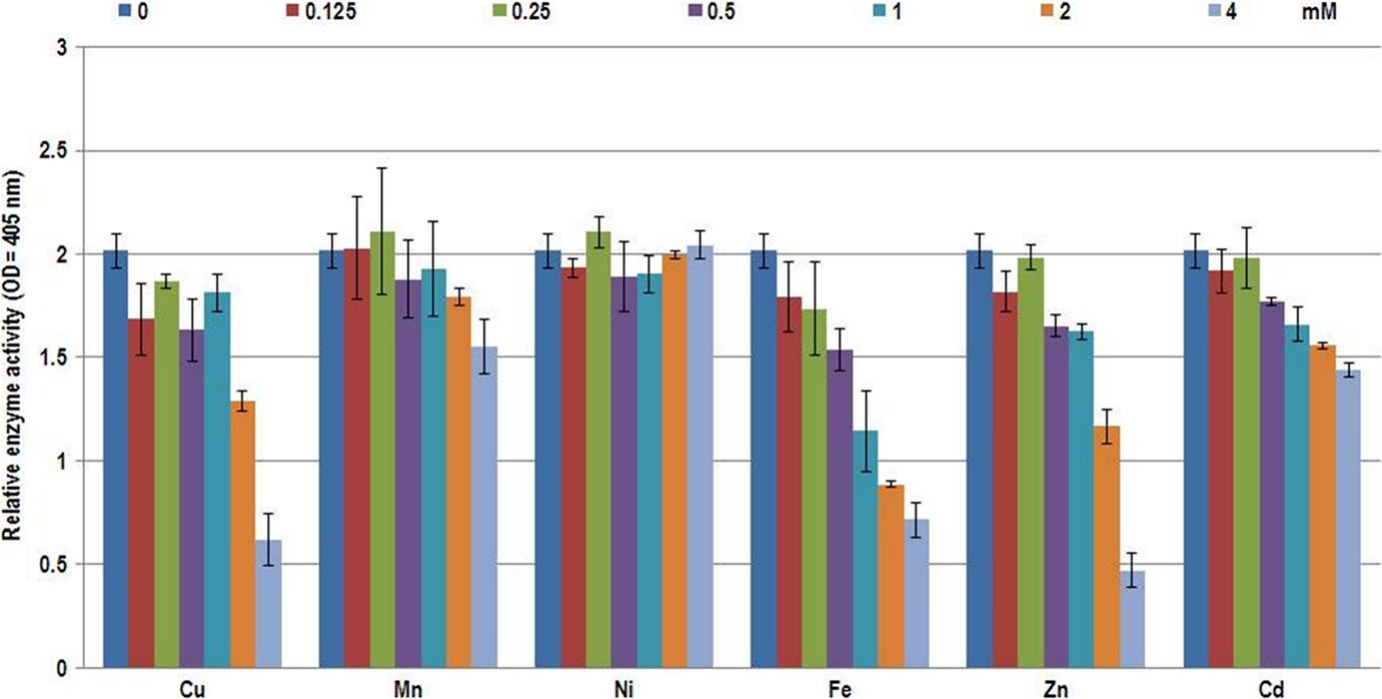
Effect of heavy metal ions on the chymotrypsin‐like protease activities on the fifth day in the cell‐free supernatant of shaken liquid culture of *B. velezensis* SZMC 6161J, produced in heavy metal‐free, liquid YEG medium

The effect of distinct metals on the activities of extracellular enzymes produced in heavy metal‐free medium was analyzed in the concentration range of 0.125–4 mmol L^−1^, where manganese and nickel ions did not influence the extracellular enzyme activities. However, at concentrations higher than 1 mmol L^−1^, copper, iron, zinc, and cadmium significantly inhibited chymotrypsin‐like protease activities (Figure 3).

The trypsin‐like protease activity in the cell‐free supernatant of shaken liquid cultures was insensitive to all tested heavy metal concentrations, rather some activation could be detected, and palmitoyl esterase was also insensitive to the investigated heavy metals in this concentration range (data not shown).

### 3.3 Effect of pesticides on strain *B. velezensis* SZMC 6161J

**Figure 4:**
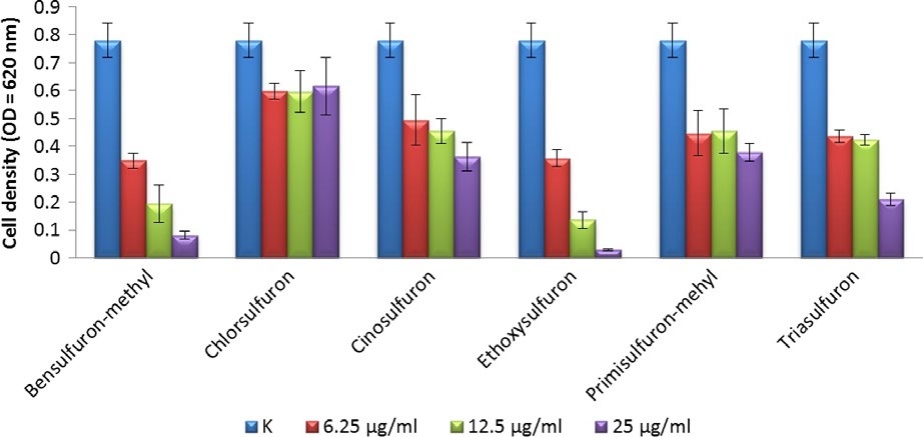
Effect of sulfonylurea herbicides on the growth of *B. velezensis* SZMC 6161J

**Figure 5:**
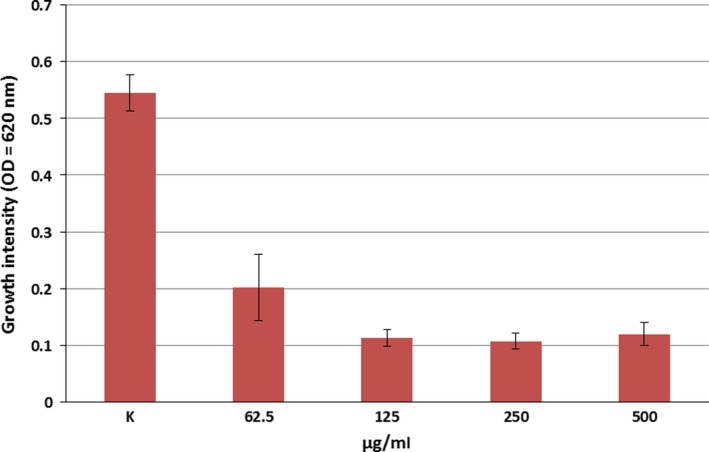
Inhibitory effect of glyphosate on the growth of *B. velezensis* SZMC 6161J

The fungicides carboxine, flutriafol, thiabendazole, and thiophanate‐methyl, as well as the herbicides chlortoluron, diuron, glyphosate, isoproturon, linuron, propham, and 2,4‐D did not inhibit the growth of strain SZMC 6161J at their concentrations of 20 mg L^−1^ (data not shown). Sulfonylurea herbicides inhibited the growth of the strain in minimal medium during the 48 hr of incubation. Bensulfuron‐methyl, cinosulfuron, ethoxysulfuron, and triasulfuron reduced the biomass production of the strain already at a concentration of 6.25 mg L^−1^, while higher concentrations of ethoxysulfuron led to the complete inhibition of bacterial growth (Figure 4). Glyphosate and 2,4‐D (two highly water‐soluble herbicides) were tested at higher concentrations. 2,4‐D was not inhibitory even at 100 mg L^−1^, while glyphosate showed a concentration‐dependent inhibition in the concentration range of 30–125 mg L^−1^ (Figure 5). At the relatively high concentration of 125 mg L^−1^, the growth inhibition was about 80% compared to control cultures. Higher concentrations did not cause higher growth inhibitions.

**Figure 6:**
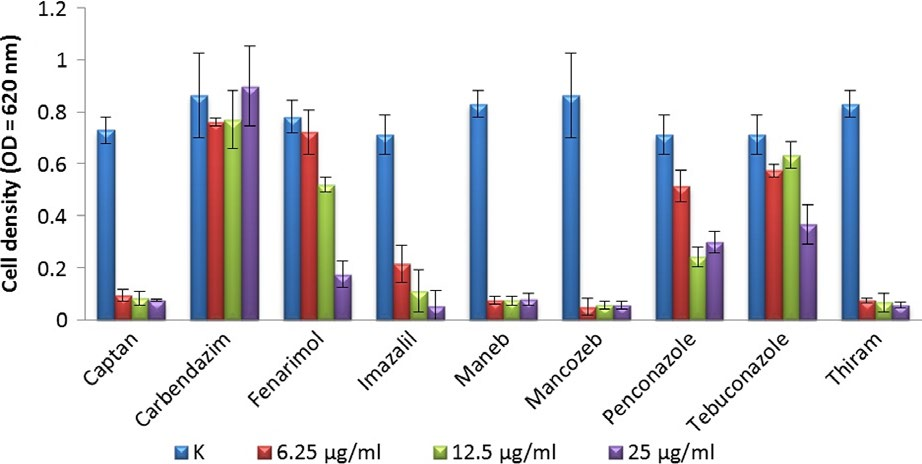
Effect of contact and azole fungicides on the growth of *B. velezensis* SZMC 6161J

Many investigated fungicides had marked inhibitory effects on the examined *Bacillus* strain. Above the concentration of 6.25 mg L^−1^, the contact fungicides captan, maneb, mancozeb, and thiram strongly inhibited the growth of the strain in a concentration‐independent manner. In contrast, demethylation‐inhibiting azole fungicides (imazalil, fenarimol, penconazole, and tebuconazole) also showed strong inhibition, but in a concentration‐dependent manner (Figure 6). Imazalil proved to be the most inhibitory among the examined azole compounds.

Carbendazim slightly inhibited bacterial cell growth in the analyzed concentration range. Higher concentrations could not be applied due to the solubility limit of this compound in water. The effect of herbicides and fungicides on the chymotrypsin‐like protease activities of the strain was tested at concentrations of 25, 12.5, and 6.25 mg L^−1^. We did not measure any inhibitory or activating effect, not even in the case of the contact fungicides.

### 3.4 Heavy metal‐ and pesticide‐tolerant mutants of strain SZMC 6161J and their characteristics

Cadmium‐, copper‐, captan‐, imazalil‐, and maneb‐tolerant spontaneous mutants were isolated at the MIC values and some cases at higher concentrations of the compounds. The frequency of the mutants in the cell population of the bacterial strain is shown in Table 1.

**Table 1:** The frequency of spontaneous, metal‐ and pesticide‐tolerant mutants in the cell population of *B. velezensis* SZMC 6161J and their MIC values

Compound	MIC values of the wild strain in liquid YEG medium (mg L^−1^)	Isolation concentrations on solid YEG medium (mg L^−1^)	Frequency of tolerant mutants (×10^−7^)	Average of MIC values of 10 mutant strains in liquid YEG medium (mg L^−1^)
CdSO_4_	0.625 ± 0.00	10^a^	3.20 ± 1.70	1.25 ± 0.05
CuSO_4_	46 ± 0.01	50	10.75 ± 1.37	72.78 ± 5.44
Captan	60 ± 0.12	60	36.25 ± 17.78	124.25 ± 3.53
Imazalil	20 ± 0.03	20	61.25 ± 13.64	42.47 ± 1.32
Maneb	25 ± 0.00	25	42.75 ± 10.58	52.16 ± 1.38

a The wild strain showed more higher tolerance on solid medium; it was able to grow at 5 mg L^−1^, so the mutant isolation occurred at 10 mg L^−1^.

In the case of all five inhibitors, 10‐10 tolerant strains were randomly selected and their exact MIC values in YEG liquid medium and their antagonistic potential on YEG solid medium was investigated against some phytopathogenic bacteria and fungi in comparison with the parental strain SZMC 6161J. The results are shown in Tables 1 and 2. The MIC values of mutants in all cases were about twice of the MIC values of the wild‐type strain (Table 1).

**Table 2:** Results of ANOVA analyses in pairwise comparison of the inhibitory activities (diameters of inhibition zones in mm) of tolerant mutants and the wild‐type strain *B. velezensis* SZMC 6161J against bacteria and fungi. Ten, randomly selected resistant mutants were investigated with all compounds. Methodology of the antagonism tests is described in the chapter 2.8

Factors			Mean difference	*SE*	*p*
*Agrobacterium radiobacter* SZMC 21783					
Wild	‐	CdR	−0.800	0.200	**0.0467**
	‐	CuR	−1.600	0.267	**0.0030**
	‐	CapR	−1.200	0.200	**0.0030**
	‐	ImaR	−0.600	0.163	0.0768
	‐	ManR	0.110	0.149	1.0000
*Xanthomonas campestris* SZMC 6182					
Wild	‐	CdR	0.250	0.250	1.0000
	‐	CuR	−0.600	0.163	0.0768
	‐	CapR	−0.100	0.180	1.0000
	‐	ImaR	0.000	0.000	
	‐	ManR	0.600	0.145	*0.0384*
*Botrytis cinerea* SZMC 21473					
Wild	‐	CdR	1.200	0.512	0.6566
	‐	CuR	−1.800	0.533	0.1229
	‐	CapR	1.900	0.526	0.0846
	‐	ImaR	1.600	0.499	0.1606
	‐	ManR	−3.000	0.494	**0.0028**
*Fusarium oxysporum* SZMC 6237J					
Wild	‐	CdR	2.600	0.306	*0.0002*
	‐	CuR	1.000	0.422	0.6269
	‐	CapR	1.550	0.217	*0.0008*
	‐	ImaR	2.050	0.217	*0.0001*
	‐	ManR	0.400	0.400	1.0000
*F. graminearum* SZMC 11037					
Wild	‐	CdR	2.300	0.423	*0.0062*
	‐	CuR	−0.400	0.371	1.0000
	‐	CapR	2.500	0.500	*0.0111*
	‐	ImaR	1.350	0.350	0.0580
	‐	ManR	−0.700	0.335	0.9934
*F. solani* SZMC 11070F					
Wild	‐	CdR	1.400	0.476	0.2471
	‐	CuR	0.100	0.180	1.0000
	‐	CapR	1.400	0.400	0.1009
	‐	ImaR	1.500	0.453	0.1366
	‐	ManR	−0.500	0.307	1.0000

*Note*. Inhibitory activities significantly (*p* < .05) better or significantly worse than that of the wild‐type strain *B. velezensis* SZMC 6161J are set in bold and italic, respectively.

CdR: cadmium‐tolerant, CuR: copper‐tolerant, CapR: captan‐tolerant, ImaR: imazalil‐tolerant, ManR: maneb‐tolerant strains.

**Figure 7:**
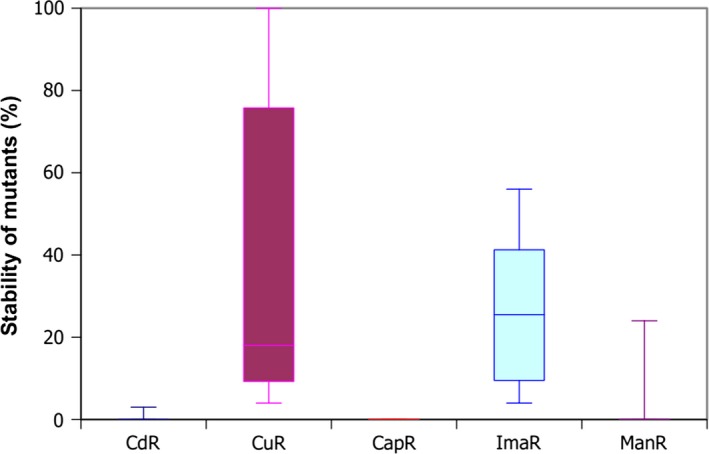
Stability of heavy metal‐ and pesticide‐tolerant mutants of *B. velezensis* SZMC 6161J culturing without selection pressure in YEG medium. Statistical box plot graph showing the distribution of the stability values of tolerant mutants was generated with the Gnumeric Spreadsheet 1.10.16 software. Abbreviations: CdR: cadmium‐tolerant, CuR: copper‐tolerant, CapR: captan‐tolerant, ImaR: imazalil‐tolerant, ManR: maneb‐tolerant strains

The stability of the mutants was also investigated and the result is presented in Figure 7 as a statistical box plot graph. Figure 7 shows that the copper‐tolerant strains show high or medium stability; imazalil‐ and maneb‐resistant strains show medium stability, but cadmium‐ and captan‐resistant mutants are very instable when cultured without selection pressure in YEG liquid medium.

The tested mutants preserved their antagonistic properties against both bacteria and fungi (Table 2). Although in some cases, the ANOVA test shows statistically significant deviations from the wild‐type values, as the deviations are low values these very likely resulted from the low reproducibility of the antagonism tests. It is very probable that the distinctions detected resulted from some fluctuation errors of the in vitro antagonism test system leading to little, but statistically significant data drift. Considering this, it seems that there is no convincing difference between the antagonism potential of the wild‐type strain and its derived resistant mutants.

## 4 DISCUSSION

BCAs are frequently applied in the rhizosphere against soil‐inhabiting plant pathogenic bacteria and fungi. However, the soils of intensive agricultural areas are frequently polluted with various heavy metals (Giller, Witter, & Mcgrath, 1998). As some of these ions are occasionally present at concentrations inhibitory to microorganisms, this pollution could highly influence the behavior of rhizobacteria (Alloway, 2013).

The worldwide situation of soil pollution with selected heavy metals is shown in Table 3. These data indicate that the concentration ranges of heavy metals used in our experiments are actually present in various agricultural soils.

**Table 3:** Heavy metals content of agricultural soils worldwide (mmol/kg)

	Cu	Mn	Ni	Fe	Zn	Cd
Average	0.60	13.1	0.45	74.5	1.81	0.013
Range (mmol/kg)	0.02–1.69	0.92–54.81	0.002–0.98	0.07–290.19	0.07–6.58	0.0004–0.12

Compiled from data published for China (Huang et al., 2007; Li et al., 2009; Su, Jiang, & Zhang, 2014), Spain (Zimakowska‐Gnoinska, Bech, & Tobias, 2000), Korea (Kim & Kim, 1999), Slovakia (Wilcke, Krauss, & Kobza, 2005), USA (Jean‐Philippe, Labbé, Franklin, & Johnson, 2012), and India (Raju, Somashekar, & Prakash, 2013) regarding copper (Cu), nickel (Ni), zinc (Zn), and cadmium (Cd) concentrations. The data for manganese (Mn) and iron (Fe) were calculated from the publications of Frank, Ishida, and Suda (1976) and Soumaré, Tack, and Verloo (2003).

Cadmium, copper, zinc, and nickel likely display inhibitory effects against microbial BCAs (Hattori, 1992; Oliveira & Pampulha, 2006; Wang et al., 2007). Furthermore, the pH of the soil in the root zone of the plants is generally slightly acidic (because of the acidic root exudates), thereby increasing the level of the soluble forms of some heavy metals (Dakora & Phillips, 2002). There are some frequently used pesticides, from which ionic forms of certain heavy metals are released (e.g., copper from copper fungicides, manganese from maneb and mancozeb, zinc from mancozeb and zineb). Moreover, cadmium is regularly introduced into the soil through phosphate fertilizers (Jiao, Chen, Chang, & Page, 2012; Nacke et al., 2013; Wu et al., 2012). Although *B. velezensis* SZMC 6161J tolerated the presence of manganese ions, low concentrations of copper, nickel, zinc, and cadmium highly reduced the growth of this bacterium. In addition, copper, iron, zinc, and cadmium lowered the activity of the secreted extracellular chymotrypsin‐like proteases, suggesting that the biocontrol application of this strain can be more efficient in the phyllosphere, where lower levels of heavy metals are present, than in the soil. The efficacy of soil applications could be increased with successor strains which tolerate heavy metals at a higher level through the overexpression of metal‐resistant enzymes (Nies, 1999). Effron et al. (2004) showed that heavy metals inhibit the activities of proteases in the soil. Interestingly, both the trypsin‐like proteases and esterases of the examined *B. velezensis* strain were tolerant to heavy metals in the tested concentration range. The activity of the trypsin‐like protease of SZMC 6161J was elevated in the presence of divalent metal ions, suggesting that this protease is a metalloprotease. A neutral metalloprotease and an alkaline serine‐protease have previously been described from the shaken liquid culture of *B. velezensis* (Cho, Oh, Pridmore, Juillerat, & Lee, 2004). Similar studies characterizing various biocontrol candidate bacteria are essential to determine the value of these microbes for soil pest control in agricultural practices.

A compilation of estimated soil concentrations for the pesticides examined in the present work is shown in Table 4. After the field dispersal, these pesticide concentration ranges are characteristic for the uppermost 20 cm of the soil. These concentration values could dynamically change, depending among others on the microbial degradability of a given compound. In certain cases the concentration value is quickly decreasing, but in dry periods it can increase again in the water phase of the rhizosphere. Values of Table 4 confirm that the pesticide tolerance testing of our *Bacillus* strain occurred in the appropriate concentration ranges.

**Table 4:** Estimated soil concentration ranges and experimental concentration ranges of the pesticides in our experiments

	Soil concentration reported earlier (mg/kg)	References	Concentration used in our experiments (mg/l)
Fungicides			
Captan	0.94–14.7	Martınez‐Toledo, Salmeron, Rodelas, Pozo, & Gonzalez‐Lopez, (1998)	6.25–25
Carbendazim	2–10	Yu, Chu, Pang, Xiang, & Fang (2009)	6.25–25
Carboxine	5–20	Mathre, Johnston, & Grey, (2001)	20
Fenarimol	0.5–10	Annex I of Directive 91/414/EEC (2007)	6.25–25
Flutriafol	10–24	Karaoglanidis, Loannidis, & Thanassoulopoulos, (2002)	20
Imazalil	5–20	Mathre et al. (2001)	6.25–25
Mancozeb	5–20	Mathre et al. (2001)	6.25–25
Maneb	5–20	Mathre et al. (2001)	6.25–25
Penconazole	1–15	Monaci, Coppola, Casucci, & Vischetti, (2011)	6.25–25
Tebuconazole	5–20	Mathre et al. (2001)	6.25–25
Thiabendazole	5–20	Mathre et al. (2001)	20
Thiram	5–20	Mathre et al. (2001)	6.25–25
Thiophanate‐methyl	1–26	Fleeker, Lacy, Schultz, & Houkom (1974)	20
Herbicides			
Bensulfuron‐methyl	0.01–10	Blair & Martin (1988)	6.25–25
Cinosulfuron	0.01–10	Blair & Martin (1988)	6.25–25
Chlorsulfuron	0.01–10	Blair & Martin (1988)	6.25–25
Chlortoluron	5–25	Sørensen, Bending, Jacobsen, Walker, & Aamand (2003)	20
Diuron	5–25	Sørensen et al. (2003)	20
Ethoxysulfuron	0.01–10	Blair & Martin (1988)	6.25–25
Glyphosate	20–200	Zobiole, Kremer, Oliveira, & Constantin, (2011)	20–500
Isoproturon	1–10	Sørensen et al. (2003)	20
Linuron	5–25	Sørensen et al. (2003)	20
Primisulfuron‐methyl	0.01–10	Blair & Martin (1988)	6.25–25
Propham	2–25	Tena, Garrido, & Magallanes, (1984)	20
Triasulfuron	0.01–10	Blair & Martin (1988)	6.25–25
2,4‐Dichlorophenoxyacetic acid (2,4‐D)	5–50	Lavy, Roeth, & Fenster, (1973)	20–100

The sulfonylurea herbicides inhibit acetolactate synthase (ALS), which can be found in plants and microorganisms and catalyzes the first step in the synthesis of the branched‐chain amino acids (Brown, 1990). Our results suggest that *B. velezensis* SZMC 6161J has a sulfonylurea‐sensitive ALS. Based on this, the coapplication of sulfonylurea herbicides and *B. velezensis* SZMC 6161J is not recommended as most of the soils and the plant surfaces are poor in branched‐chain amino acids which could suppress this inhibitory effect. Inhibitory effect of sulfonylurea herbicides on some soil‐inhabiting (Arabet et al., 2014) and pathogenic bacteria (Kreisberg et al., 2013) were already reported.

Glyphosate inhibited *B. velezensis* SZMC 6161J above the concentration of 30 mg L^−1^. This herbicide acts at the aromatic amino synthesis pathway by inhibiting the enzyme 5‐enolpyruvylshikimate‐3‐phosphate synthase (EPSPS), which catalyzes the reaction of shikimate‐3‐phosphate (S3P) and phosphoenolpyruvate to form 5‐enolpyruvylshikimate‐3‐phosphate (EPSP) both in plants and microorganisms (Steinrücken & Amrhein, 1980). In this way, glyphosate could cause dramatical changes in microbial diversity of soils at the treated fields (Busse, Ratcliff, Shestak, & Powers, 2001; Ratcliff, Busse, & Shestak, 2006).

The interesting phenomenon that azole‐type fungicides could be inhibitory to some bacteria has been observed in the case of the genera *Mycobacterium* and *Streptomyces* (McLean et al., 2002). It is suggested that this sensitivity is connected to the presence and interaction with some cytochrome P450 monooxygenases, which are also present in the members of the genus *Bacillus* (Furuya, Shibata, & Kino, 2009). It is known from *B. subtilis* that a cytochrome, P450 BioI (CYP107H1) is involved in the early stages of biotin synthesis and this monooxygenase can hydroxylate essential fatty acids. As the *B. subtilis* P450 BioI strongly binds steroids and azole compounds (Lawson et al., 2004), it can be presumed that these are the targets of inhibition by azoles in the examined *B. velezensis* strain.

Our results show that it is crucial to determine the heavy metal and pesticide tolerance of a biocontrol candidate microorganism before application in IPM cultivation systems. If a strain is highly sensitive to a heavy metal or a pesticide, tolerant mutants can be isolated. We also proved that tolerant strains with preserved biocontrol potential could be isolated without any mutagenic treatment, as the spontaneously occurring tolerant cells are already present in the cell population of the bacterium with a frequency of 10^−6^–10^−7^.

## CONFLICT OF INTEREST

The authors declare that the research was conducted in the absence of any commercial or financial relationships that could be construed as a potential conflict of interest.
